# The Role of Fresh Beef Intake and Mediterranean Diet Adherence during Pregnancy in Maternal and Infant Health Outcomes

**DOI:** 10.3390/nu16101436

**Published:** 2024-05-09

**Authors:** Danielle Nicole Christifano, Kathleen M. Gustafson, Susan E. Carlson, Nicole B. Mathis, Alexandra Brown, Obianuju Onuoha, Matthew K. Taylor

**Affiliations:** 1 Department of Dietetics and Nutrition, University of Kansas Medical Center, Kansas City, KS 66160, USA; scarlson@kumc.edu (S.E.C.); nmathis@kumc.edu (N.B.M.); oonuoha@kumc.edu (O.O.); mtaylor3@kumc.edu (M.K.T.); 2 Hoglund Biomedical Imaging Center, University of Kansas Medical Center, Kansas City, KS 66103, USA; kgustafson@kumc.edu; 3 Department of Neurology, University of Kansas Medical Center, Kansas City, KS 66160, USA; 4 Department of Biostatistics and Data Science, University of Kansas Medical Center, Kansas City, KS 66160, USA; abrown8@kumc.edu

**Keywords:** Mediterranean diet, beef, anemia

## Abstract

Beef is an excellent source of nutrients important for maternal health and fetal development. It is also true that the Mediterranean diet is beneficial for the health of both the mother and offspring; however, the relative value of fresh beef intake within Mediterranean diet patterns during pregnancy is unknown. The objective of this project was two-fold: (1) assess the relationship between beef intake and nutrient intake in a pregnant population; (2) assess the relationship between maternal beef consumption among varying degrees of Mediterranean diet adherence with maternal risk of anemia and infant health outcomes. This is a secondary analysis of an existing cohort of pregnant women (*n* = 1076) who participated in one of two completed clinical trials examining the effect of a docosahexaenoic acid supplementation on birth and offspring outcomes. Women were enrolled between 12 and 20 weeks of gestation and were followed throughout their pregnancies to collect maternal and infant characteristics, food frequency questionnaires [providing beef intake and Mediterranean diet (MedD) adherence], and supplement intake. Women with the highest fresh beef intake had the highest intake of many micronutrients that are commonly deficient among pregnant women. Fresh beef intake alone was not related to any maternal or infant outcomes. There was a reduced risk of anemia among women with medium to high MedD quality and higher fresh beef intake. Women in the medium MedD group had 31% lower odds of anemia, and women in the high MedD group had 38% lower odds of anemia with every one-ounce increase in fresh beef intake, suggesting that diet quality indices may be misrepresenting the role of fresh beef within a healthy diet. These findings show that beef intake increases micronutrient intake and may be protective against maternal anemia when consumed within a healthy Mediterranean diet pattern.

## 1. Introduction

Beef is an excellent source of macro and micronutrients (e.g., protein, iron, vitamin B12, vitamin B6, choline, zinc), which are commonly deficient in the diets of pregnant women in the United States [1] yet are crucial for maternal health and fetal growth and development. Beef is an especially good source of iron as it exists in a form (i.e., heme iron) that is superior for absorption and contributes more than 10% of total absorbed iron [2]. Compared to a non-pregnant state, iron needs to increase 1.5-fold per day in pregnancy to account for the increase in maternal blood volume to meet the needs of the developing fetus [3]. If iron needs are not met during pregnancy, there is a greater risk of developing iron deficiency anemia, a condition that can result in complications during pregnancy for both the mother and the infant [3].

According to the US Preventive Services Task Force, iron deficiency is a significant problem in the United States, affecting approximately 18% of pregnant women [4]. While a modest decrease in hemoglobin levels is expected during pregnancy due to the increased blood volume, the inability to achieve replete iron status can result in an increased risk of maternal illness, intrauterine growth restriction, low birthweight, and preterm birth [5,6]. Iron supplementation has been shown to reduce the rate of iron deficiency anemia in the short term; however, it is less clear how iron supplementation in pregnancy is related to maternal and infant health outcomes [4]. As with all nutrients, iron does not work in isolation to affect obstetric health outcomes. Rather, it is a combination of factors and the synergy of nutrients present in the diet (e.g., calcium, ascorbic acid, polyphenols, phytic acid, etc.) that work together to affect iron status and subsequent health outcomes [7]. For this reason, assessing iron intake in the context of iron-rich food (beef) and healthful dietary patterns may reveal connections to maternal health risks that are not as evident when looking only at supplementation.

One such dietary pattern that has been studied in terms of its health benefits for the mother and infant is the Mediterranean diet [8]. Components of a traditional Mediterranean diet include vegetables, fruits, whole grains, nuts, legumes, seafood, and olive oil [8]. A recent systematic review reported that the Mediterranean diet was effective at reducing the risk of gestational diabetes, pregnancy-induced hypertension, intrauterine growth restriction, small for gestational age, and preterm birth [9]. The Mediterranean diet quality index (MedD) is a reliable tool for measuring adherence to a diet rich in fruits, vegetables, whole grains, and fish [10]. Although some Mediterranean diets include regular consumption of red meat [11], the index score deducts points for red meat intake, citing meat as a “non-traditional” food [10]. However, the existing literature regarding maternal diet patterns [8,12] does not differentiate between fresh beef (a nutrient-dense food) and processed meat (a possible detriment to health due to the use of different processing methods and additives) [13], resulting in conflicting recommendations regarding consumption of beef during pregnancy. Further, the variation in the definition of different meat types and its composition prior to now have impacted recommendations for beef consumption [14,15].

To gain insight into beef intake in pregnancy, we leveraged two large randomized clinical trials involving pregnant women [16,17] to examine (1) the relationship between maternal beef consumption and macro and micronutrient intake and (2) the relationship between maternal beef intake, Mediterranean diet adherence, and maternal and infant health. Within the second objective, we aimed to determine if maternal fresh beef intake (g/day) alone was associated with a lower risk of maternal anemia and if maternal fresh beef consumption within varying levels of Mediterranean diet pattern adherence was associated with a lower risk of anemia and better infant health (i.e., greater gestational age at delivery and acceptable for gestational age (AGA) birthweight).

## 2. Methods

### 2.1. Participants

Data from 1076 unique participants who participated in one of two recently completed clinical trials (PANDA: NCT0270239 and ADORE: NCT02626299) that examined the effect of docosahexaenoic acid (DHA) supplementation on fetal neurodevelopment (PANDA), infant cognitive outcomes (PANDA), and risk of preterm birth (ADORE) were available for this secondary analysis. The parent trials were conducted in the Midwestern United States at sites in Kansas and Ohio. The primary outcomes for both trials have been previously published [16,17]. For this secondary analysis, we excluded *n* = 238 for implausible energy intake based on previously established energy intake ranges in pregnancy [18] (<1075 or >4777 kcals/day), which provided *n* = 838 for the analyses reported in this paper. In both studies, women were enrolled between 12 and 20 weeks of gestation and were followed closely throughout their pregnancies to collect maternal characteristics, infant characteristics, food frequency questionnaires, and supplement intake. All data collection procedures relevant to this study are outlined in the following sections. Of note, blood samples were collected for the parent clinical trials to measure fatty acid content at baseline and delivery; however, there were no blood metrics used when assessing outcomes in this secondary analysis.

### 2.2. Maternal and Infant Characteristics

Maternal: Demographic (age, race/ethnicity), anthropometric (weight, height, BMI), and pertinent medical history (hemoglobin levels) were collected from the participant’s medical record at baseline and throughout pregnancy. Diagnosis of anemia was documented based on widely accepted cutpoints and confirmed in the medical record [19].

Infant: Gestational age at birth, birthweight, length, and head circumference were recorded from the infants’ medical records. Infant size was categorized as small (SGA), acceptable (AGA), or large (LGA) for gestational age using WHO standards [20].

### 2.3. Food Frequency Questionnaire

Participants completed the National Cancer Institute Diet History Questionnaire II (DHQ-II) [21] between 12 and 20 weeks of gestation via an online portal on a secure website. Participants were given the option to complete the questionnaire on a study team tablet while at the clinic or to complete it at home using their home internet. The database associated with the DHQ versions is based on the National Health and Nutrition Examination Surveys data collection from 2001 to 2006. In this study, participants completed the version of the survey that captured their diet over the past year with portion sizes. DHQ-II data were exported at the end of this study, and data were used to calculate beef intake and Mediterranean diet adherence.

### 2.4. Supplement Intake

Supplemental iron intake was collected through a dietary supplement inventory at baseline and monthly throughout their pregnancy to determine supplement type, dosage, and frequency of consumption. When possible, web links to supplement brands or photos of supplements were collected to verify the specific nutrient content of each reported nutrient. Iron consumed from prenatal vitamins or other supplements was included in the total supplemental iron intake. Total iron consumed via supplements was calculated as average grams per day throughout pregnancy.

### 2.5. Beef Intake

The DHQ-II Diet*Calc 1.5.0 [22] software was used to export the “Detailed Analysis File”, which contained the average daily intake for each of the individual foods included in the DHQ-II survey. We calculated the daily average of total fresh beef intake in ounces by summing the daily average intake of ground beef, roasts, and steaks either as a single food item or within a mixed dish. Processed meat was not included in this calculation.

### 2.6. Mediterranean Diet Adherence

The DHQ-II Diet * Calc software was also used to export files for the calculation of Mediterranean diet pattern scores (MedD). MedD scores were calculated for each participant using a modified 18-point Sofi et al. Mediterranean Diet Index [10,23]. To elucidate the role of fresh beef within the MedD pattern with maternal and fetal outcomes, we removed the red meat score, a score that is higher with avoidance of red meat, from the MedD index calculation, reducing the maximum score to 16. We then calculated MedD adherence tertiles to characterize participants as low, medium, and high MedD adherers according to methods published previously [23,24].

### 2.7. Statistical Analysis

We performed descriptive statistics by presenting mean ± standard deviation (SD) of nutrient intake among all participants and within fresh beef intake tertile groups. The mean contrast of nutrient intake among fresh beef tertile groups was performed using a one-way analysis of variance (ANOVA). We also contrasted mean MedD adherence scores and total fresh beef intake among MedD adherence groups.

We constructed ordinary least squares (OLS) regression models to test whether fresh beef intake (g/day) was related to infant (gestational age at birth, length, head circumference) health variables. We also constructed a binary generalized linear model (GLM) to assess whether fresh beef intake was associated with increased odds for AGA. For additional interpretation using clinically relevant variables, we constructed additional binary GLM models to assess whether a higher intake of fresh beef was associated with lower odds for clinical diagnosis of iron deficiency anemia. We included age, race/ethnicity, supplemental iron intake, pre-pregnancy BMI, and DHA status at delivery as covariates in all models.

We also constructed nested OLS regression models to test whether the relationships of total fresh beef intake with maternal and infant health (hemoglobin, gestational age at birth, length, head circumference, and AGA) were different among low, medium, and high MedD diet adherence. To test whether beef intake was associated with different odds for clinical diagnosis of anemia among low, medium, and high MedD adherence, we constructed binary GLM models with a similar nested interaction term of beef intake within the MedD adherence group. All model assumptions were assessed using residual analyses (quantile–quantile plots, histograms, and scale–location plots). Age, race/ethnicity, pre-pregnancy BMI, supplemental iron intake, and DHA status were included as covariates in all models.

## 3. Results

Sample characteristics are shown in Table 1, and nutrient intake among tertiles of beef consumers is shown in Table 2. Women in tertile 3 who consumed the highest amount of beef (mean ± SD: 1.5 ± 0.7 oz/day) consumed significantly more energy in calories, protein, fat (mono, poly, and saturated), and carbohydrates relative to women in the medium and low beef groups. Notably, tertile 3, with the highest beef consumption, was the only group to have a mean protein intake that met the Recommended Daily Allowance (RDA) of 71 g/day for protein in pregnancy. In terms of micronutrients, women in tertile 3 consumed significantly higher quantities of vitamins (A, thiamin, riboflavin, niacin, B6, folate, B12, choline) and minerals (calcium, phosphorus, magnesium, iron, zinc, copper, selenium, sodium, potassium) from their diet. However, there was no significant difference in vitamin C and K intake by tertile of beef consumption. Supplemental iron intake among the sample was 19.0 ± 12.9 mg/day and did not differ across the tertiles of beef intake or MedD adherence.

Fresh beef intake alone was not related to any maternal or infant outcomes when accounting for covariates (Table 3). There was neither statistical significance between maternal fresh beef intake and anemia (*p* = 0.11) nor hemoglobin (*p* = 0.12). Again, our study did not show any statistical significance between maternal fresh beef intake and gestational age at birth, length, head circumference, and AGA. To examine how beef intake within the context of a Mediterranean diet affected maternal and infant health outcomes, we first assessed mean MedD adherence and total beef intake among low, medium, and high MedD adherence groups. Out of a maximum possible score of 16, the MedD scores were as follows: low: 2.1 ± 0.8; medium: 4.0 ± 0.7; and high: 6.4 ± 1.2 (*p* < 0.001). Mean intakes and variability of fresh beef were similar among MedD adherence groups with daily intake of low: 0.8 ± 0.7 oz., medium: 0.7 ± 0.6 oz., and high: 0.7 ± 0.7 oz (*p* = 0.37).

**Figure 1:**
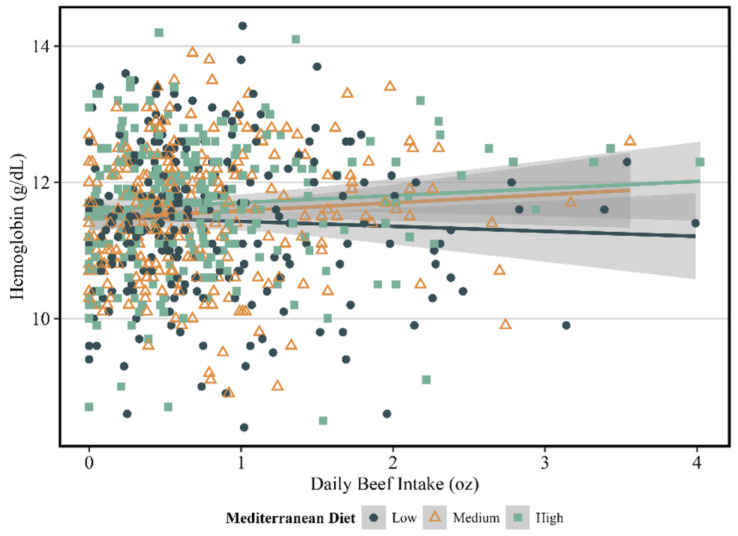
Relationship between fresh beef intake and maternal hemoglobin among differing levels of adherence to the MedD.

Higher fresh beef intake was related to maternal hemoglobin levels at mid-pregnancy in a way that was influenced by MedD adherence, such that women with a higher beef intake accompanied with high MedD adherence had statistically significant higher hemoglobin levels (beta = 0.19; SE = 0.08; *p* = 0.01) (Table 4, Figure 1). None of the infant outcomes of interest showed any significant relationship between fresh beef intake and the different levels of Mediterranean diet adherence.

There was a similar trending relationship among women with medium MedD adherence (beta = 0.13; SE = 0.08; *p* = 0.10). Beef intake among women in the low MedD group was not related to hemoglobin levels (beta = −0.09; SE = 0.08; *p* = 0.27). From a clinical perspective, 36% of participants met the criteria for anemia. Higher beef intake in the low MedD group was not related to lower odds of anemia; however, women in the medium MedD group had 31% and significantly lower odds of anemia (OR: 0.69; 95% CI: 0.46–0.99; *p* = 0.05). Also, women in the high MedD group had 38% and significantly lower odds of anemia (OR: 0.62; 95% CI: 0.41–0.89; *p* = 0.01) with every one-ounce increase in fresh beef intake (Table 5). Higher adherence to the MedD alone was not related to lower odds of anemia (OR: 0.98; 95% CI: 0.90–1.06; *p* = 0.62). No other relationships were found between diet quality and beef intake and maternal or infant outcomes.

## 4. Discussion

We examined if consumption of maternal intake of fresh beef intake was related to increased intake of micronutrients in pregnancy and whether beef intake, alone and in the context of a healthy Mediterranean diet, was related to maternal and infant health outcomes. Our results show that increased beef intake was related to increased energy and macro and micronutrient intake. Women who ate the most beef were the closest to meeting the Estimated Average Requirements (EAR) for several important nutrients commonly deficient in pregnant populations, such as protein, iron, vitamin B12, vitamin B6, choline, and zinc. Our finding agrees with a review [25], which posits that adequate meat intake accompanied by a healthy diet is beneficial, especially during increased needs like pregnancy. While beef intake alone was not associated with health outcomes (positive or negative), increased beef intake within a healthier diet was related to higher hemoglobin levels in mid-pregnancy and lower risk of anemia. This aligns with a study conducted in non-pregnant individuals in the United Kingdom, where lower or no red meat intake was associated with a higher risk of anemia and lower hemoglobin levels [26]. These studies align with our findings, suggesting that lower or no beef intake during pregnancy could be a risk factor for developing anemia.

There are two major implications of this work: one for the future of diet quality research in pregnancy; and the other for dietary advice to reduce the risk of anemia for pregnant women. In terms of diet quality research in pregnancy, it may be warranted to reevaluate whether it is appropriate for red meat to be associated with negative scores in diet quality score calculation. When we excluded red meat calculations from the Mediterranean diet quality index score, we found a reduced risk of anemia among women with medium to high diet qualities and higher beef intakes, suggesting that diet quality indices may be misrepresenting the role of beef within a healthy diet. Our work is in line with other studies that found no increased risks but possible benefits of including red meat as part of a healthy Mediterranean diet pattern among individuals at high risk for cardiometabolic disease [27].

Second, our findings suggest that a higher fresh beef intake rather than low intake or total exclusion from the diet may be protective against anemia and could be recommended as a nutrient dense food source to be encouraged in a prenatal diet. Recommending whole food intake in pregnancy is a reasonable approach to increasing micronutrient intake and reducing health risks among pregnant women and their infants. Prenatal supplements are widely recommended in pregnancy; however, many women report issues with inadequate support, accessibility, and overwhelming surrounding supplementation in ways that affect their adherence [28,29]. Women who inconsistently take prenatal vitamins, including iron supplements, report being skeptical of the efficacy, experiencing adverse effects such as gastrointestinal discomfort, and issues with access to supplements during pregnancy [29]. Thus, recommending a whole food, such as beef, that is well tolerated, readily available at most grocery stores, and reasonably priced is a reasonable strategy for increasing micronutrient and macronutrient intake in pregnancy.

Research in non-pregnant populations has examined the role of red meat in the diets of infants, adolescents, women of reproductive age, and older adults as it relates to cardiometabolic, cognitive, and cancer-related outcomes. The American Institute for Cancer Research suggests limiting red meat intake to less than 18 oz per week due to an increased risk of colorectal cancer. This recommendation is based largely on epidemiological data showing excessive intake of red meat, processed meat, and iron and higher odds of colorectal cancer [30]; however, the mechanisms driving these associations are largely unknown; the demographic group tends to err on the side of older adults [31]. Infants and women of reproductive age tend to benefit the most from beef intake due to its ability to provide nutrients important to the growth and development of the fetus and/or infant, influencing health during childhood and later in life. We are aware of no research to date that associates negative outcomes with beef intake in these groups.

The strengths of this work include a large sample size of pregnant women who reported reliable dietary intake data throughout their pregnancy and the ability to associate diet with health outcomes of interest. Further, this study highlights the inclusion of fresh beef intake as a part of a healthy diet and its potential benefits, especially in a crucial life stage such as pregnancy. This work highlights the need for clarity in differentiating beef types and presents evidence to challenge the negative perception associated with beef consumption.

Some limitations of this work include the use of dietary history questionnaires, which did not allow for the characterization of the leanness of fresh beef consumed by participants. We were limited in using the DHQ-II to capture iron intake and will pursue more specific tools to assess iron intake in future research. We were also unable to calculate a variable for total processed beef intake as the DHQ-II processed meat intake variables did not differentiate the source of intake (i.e., pork, beef, poultry) for all processed meats. Future work should use methods that account for these limitations.

## 5. Conclusions

In conclusion, beef is a rich source of macro and micronutrients that are essential for maternal health and infant growth and development. The findings from our study on beef intake and Mediterranean diet adherence during pregnancy indicate that beef intake within the context of a healthy diet could be beneficial for reducing the risk of maternal anemia and could be recommended as a healthful food in pregnancy. Also, we have demonstrated through this study that beef consumption during pregnancy is positively related to increased macro and micronutrient intake. Therefore, inadequate intake or absolute exclusion of beef from a healthy diet may not be recommendable. The results of this work challenge the negative perception often associated with beef consumption and underscore the importance of considering and encouraging beef as part of a healthy prenatal diet. Furthermore, this work clearly highlights the need to redefine various meat types and their contents accordingly to enable appropriate recommendations required by different population groups. Advocating for healthy diet quality along with whole-food sources of nutrients, like beef, may offer a practical and effective strategy for addressing nutritional deficiencies during pregnancy and subsequently promote optimal maternal and infant health.

## Figures and Tables

**Table 1:** Sample Characteristics (*n* = 838).

Maternal Characteristics	Mean ± SD
Age	30.1 ± 5.4
Pre-Pregnancy BMI	27.7 ± 7.1
Race and Ethnicity	
White	573 (68.4%)
Black or African American	156 (18.6%)
Hispanic	46 (5.5%)
Asian	30 (3.6%)
Biracial: Black, White	10 (1.2%)
Biracial: Asian, White	7 (0.8%)
American Indian or Alaskan Native	5 (0.5%)
Multiracial: Black, Native American, White	3 (0.4%)
Other	3 (0.4%)
Native Hawaiian or Other Pacific Islander	2 (0.2%)
Biracial: Native American, White	1 (0.1%)
Biracial: Black, Asian	1 (0.1%)
Multiracial: Black, Asian, White	1 (0.1%)
Anemia Diagnosis	298 (35.6%)
Hemoglobin at ~20 Weeks	11.6 ± 1.0
**Infant Characteristics**	
Sex, Female	395 (49.2%)
Gestational Age at Birth, Weeks	38.4 ± 1.9
Acceptable Gestational Age	647 (81.2%)
Birth Weight, g	3309.6 ± 560.7
Birth Length, cm	50.1 ± 3.9
Head Circumference, cm	34.1 ± 2.0

**Table 2:** Nutrient intake by tertile of beef consumption.

	All	Tertile 1: Beef Intake	Tertile 2: Beef Intake	Tertile 3: Beef Intake	*p*-Value
	*n* = 838	(*n* = 280)	(*n* = 279)	(*n* = 279)	
Energy (kcal/d)	1996.2 ± 753.3	1803.6 ± 712.5	1842.3 ± 653.9	2343.4 ± 767.6	<0.001
Total fat (g/d)	79.1 ± 31.9	66.9 ± 28.7	76.3 ± 27.4	94.2 ± 33.2	<0.001
Carbohydrates (g/d)	251.6 ± 132.2	243.9 ± 143.5	222.1 ± 110.3	288.9 ± 132.2	<0.001
Protein (g/d)	71.6 ± 28.6	59.5 ± 25.3	67.9 ± 23.7	87.5 ± 29.0	<0.001
Saturated fat (g/day)	26.4 ± 11.8	21.8 ± 10.2	25.2 ± 9.8	32.4 ± 12.7	<0.001
Monounsaturated fat (g/d)	30.3 ± 12.5	25.5 ± 11.4	29.5 ± 11.0	35.8 ± 12.8	<0.001
Polyunsaturated fat (g/d)	15.4 ± 7.0	13.7 ± 6.9	14.9 ± 6.6	17.7 ± 7.0	<0.001
Dietary fiber (g/d)	17.7 ± 8.4	16.7 ± 8.3	17.3 ± 8.2	19.2 ± 8.5	0.001
Retinol (mcg/d)	468.1 ± 262.4	436.3 ± 288.0	441.7 ± 235.3	526.5 ± 252.0	<0.001
Vitamin E (mg/d)	9.2 ± 4.2	8.7 ± 4.2	9.1 ± 4.3	9.9 ± 3.9	0.003
Vitamin K (mcg/d)	200.4 ± 216.6	208.3 ± 224.6	194.8 ± 202.1	198.2 ± 222.9	0.75
Vitamin C (mg/d)	141.2 ± 132.7	152.3 ± 171.4	123.4 ± 108.0	147.9 ± 106.8	0.02
Thiamin (B1) (mg/d)	1.5 ± 0.6	1.4 ± 0.6	1.4 ± 0.6	1.7 ± 0.6	<0.001
Riboflavin (B2) (mg/d)	2.3 ± 1.1	2.2 ± 1.1	2.2 ± 0.9	2.6 ± 1.2	<0.001
Niacin (mg/d)	22.7 ± 12.4	19.9 ± 12.0	21.0 ± 9.1	27.1 ± 14.4	<0.001
Vitamin B6 (mg/d)	2.2 ± 1.3	2.1 ± 1.3	2.0 ± 1.0	2.6 ± 1.5	<0.001
Folate (mcg/d)	425.6 ± 187.8	410.6 ± 197.7	407.8 ± 180.6	458.6 ± 180.8	0.002
Vitamin B12 (mg/d)	5.1 ± 3.1	4.4 ± 2.9	4.6 ± 2.3	6.4 ± 3.4	<0.001
Calcium (mg/d)	1153.3 ± 571.9	1112.9 ± 613.2	1079.3 ± 536.8	1267.6 ± 546.9	<0.001
Phosphorous (mg/d)	1271.4 ± 510.2	1127.3 ± 477.3	1205.5 ± 471.3	1482.0 ± 513.1	<0.001
Magnesium (mg/d)	329.7 ± 126.1	311.3 ± 120.3	321.2 ± 128.5	356.8 ± 125.2	<0.001
Iron (mg/d)	14.3 ± 6.0	13.0 ± 5.9	13.4 ± 5.6	16.4 ± 5.8	<0.001
Zinc (mg/d)	11.4 ± 4.6	9.7 ± 4.2	10.8 ± 4.1	13.9 ± 4.4	<0.001
Copper (mg/d)	1.5 ± 0.6	1.4 ± 0.6	1.5 ± 0.6	1.7 ± 0.6	<0.001
Selenium (mcg/d)	93.7 ± 38.8	78.9 ± 36.4	89.9 ± 33.1	112.3 ± 39.2	<0.001
Sodium (mg/d)	3010.3 ± 1158.3	2569.4 ± 1061.7	2841.7 ± 966.4	3621.5 ± 1172.6	<0.001
Potassium (mg/d)	2933.4 ± 1190.3	2740.3 ± 1162.6	2768.8 ± 1095.4	3291.9 ± 1231.2	<0.001
Choline (mg/d)	317.2 ± 129.5	274.8 ± 125.0	299.4 ± 106.8	377.7 ± 132.7	<0.001
Total Fresh Beef (oz/day)	0.8 ± 0.7	0.2 ± 0.1	0.6 ± 0.1	1.5 ± 0.7	<0.001

**Table 3:** Relationship between maternal fresh beef intake and maternal and infant outcomes.

	Unstandardized-Beta	Standard Error	*p*-Value
**Maternal Outcomes**			
Anemia ^a^	−0.22	0.14	0.11
Hemoglobin (~20 wks)	0.09	0.06	0.12
**Infant Outcomes**			
Gestational age at birth	−0.13	0.10	0.20
Length	0.21	0.23	0.36
Head circumference	−0.06	0.12	0.63
AGA ^a^	0.20	0.17	0.25

^a^ Binomial distribution. Ordinary least squares regression adjusted for all covariates: age; race/ethnicity; pre-pregnancy BMI; supplemental iron intake; and DHA status.

**Table 4:** Relationship between fresh beef intake (continuous variable) with MedD adherence level and maternal and infant outcomes.

	Total Beef with LowMedD			Total Beef with Medium MedD			Total Beef with High MedD		
	Beta	SE	*p*-Value	Beta	SE	*p*-Value	Beta	SE	*p*-Value
**Maternal Outcomes**									
Hemoglobin (~20 wks)	−0.09	0.08	0.30	0.13	0.08	0.10	0.19	0.08	0.01
**Infant Outcomes**									
Gestational age at birth	−0.20	0.13	0.13	−0.19	0.14	0.18	−0.01	0.13	0.91
Length	0.06	0.20	0.76	−0.05	0.22	0.83	0.28	0.20	0.17
Head circumference	−0.08	0.15	0.59	−0.07	0.16	0.67	−0.01	0.15	0.92
AGA ^a^	0.13	0.22	0.56	0.22	0.24	0.35	0.26	0.24	0.28

^a^ Binomial distribution. Ordinary least squares or binomial regression adjusted for all covariates: age; race/ethnicity; pre-pregnancy BMI; supplemental iron intake; and DHA status. Multiplicative interaction using both continuous Mediterranean diet scores and total beef intake as independent variable among all participants—hemoglobin (b = 0.07, SE = 0.03, *p* = 0.01), gestational age at birth (b = −0.08, SE = 0.04, *p* = 0.07), length (b = 0.05, SE = 0.04, *p* = 0.27), head circumference (b = −0.03, SE = 0.05, *p* = 0.51), AGA (b = 0.04, SE = 0.06, *p* = 0.50).

**Table 5:** Relationship between fresh beef intake (as a continuous variable) with MedD adherence level and risk of maternal anemia.

Predictor	Odds Ratio	95% CI	*p*-Value
Total Beef (oz): Low MedD	1.15	0.81–1.64	0.44
Total Beef (oz): Medium MedD	0.69	0.46–0.99	0.05
Total Beef (oz): High MedD	0.62	0.41–0.89	0.01

Odds ratios calculated by binary logistic regression adjusted for all covariates: age; race/ethnicity; pre-pregnancy BMI; supplemental iron intake; and DHA status. Multiplicative interaction using both continuous Mediterranean Diet scores and total beef intake as independent variable among all participants (OR = 0.86; CI = 0.76–0.79; *p* = 0.01).

## Data Availability

Data are contained within the article.
